# Brain glutamate in anorexia nervosa: a magnetic resonance spectroscopy case control study at 7 Tesla

**DOI:** 10.1007/s00213-016-4477-5

**Published:** 2016-12-01

**Authors:** Beata R. Godlewska, Alexandra Pike, Ann L. Sharpley, Agnes Ayton, Rebecca J. Park, Philip J. Cowen, Uzay E. Emir

**Affiliations:** 1 Department of Psychiatry, University of Oxford, Warneford Hospital, Oxford, OX3 7JX UK; 2 Oxford Health NHS Foundation Trust, Oxford, OX1 7JX UK; 3 Oxford Centre for Functional MRI of the Brain, Nuffield Department of Clinical Neurosciences, University of Oxford, John Radcliffe Hospital, Oxford, OX3 9DU UK

**Keywords:** Anorexia nervosa, Glutamate, Glutamine, Magnetic resonance spectroscopy

## Abstract

**Rationale:**

Anorexia nervosa (AN) is a serious psychiatric disorder with high morbidity and mortality. There are no established pharmacological treatments and the neurobiology of the condition is poorly understood. Previous studies using magnetic resonance spectroscopy (MRS) have shown that AN may be associated with reductions in indices of brain glutamate; however, at conventional field strengths (≤3 T), it is difficult to separate glutamate from its precursor and metabolite, glutamine.

**Objectives:**

The objective of the present study was to use high field (7 T) MRS to measure concentrations of glutamate, in three separate brain voxels, in women with AN.

**Methods:**

We studied 13 female participants with AN and 12 healthy female controls who underwent MRS scanning at 7 T with voxels placed in anterior cingulate cortex, occipital cortex and putamen. Neurometabolites were calculated using the unsuppressed water signal as a reference and corrected for individual cerebrospinal fluid concentration in the voxel.

**Results:**

We found that participants with AN had significantly lower concentrations of glutamate in all three voxels (mean reduction 8%, *p* = 0.002) but glutamine levels were not altered. Concentrations of N-acetylaspartate, creatine, GABA and glutathione were also unchanged. However, inositol was lower in AN participants in anterior cingulate (*p* = 0.022) and occipital cortex (*p* = 0.002).

**Conclusions:**

Women with AN apparently have widespread reductions in brain glutamate. Further work will be needed to assess if this change has pathophysiological relevance or whether it is a consequence of the many physical changes produced in AN by food restriction.

**Electronic supplementary material:**

The online version of this article (doi:10.1007/s00213-016-4477-5) contains supplementary material, which is available to authorized users.

## Introduction

Anorexia nervosa (AN) is a serious mental illness with a high morbidity and mortality. The characteristic feature of AN is an overwhelming fear of gaining weight coupled with strict measures to reduce net calorie intake; however, emotional and cognitive disturbances commonly accompany the illness (Fairburn and Harrison 2003; Zipfel et al. 2015). The best current treatments emphasise weight restoration and specific psychotherapies but outcomes are often unsatisfactory, particularly in adult patients (Smink et al. 2012). Antidepressant medication is widely used in AN but has no specific effect on the core disorder and is often unhelpful in relieving depression (Aigner et al. 2011). The development of effective pharmacological treatments for AN requires a better knowledge of underlying neurobiology (Zipfel et al. 2015).

Glutamate is the main excitatory neurotransmitter in brain and is implicated in a number of psychiatric disorders relevant to AN, including depression and obsessive compulsive disorder (Yüksel and Öngür 2010; Naaijen et al. 2015). It is possible to measure glutamate concentrations in the brain non-invasively with magnetic resonance spectroscopy (MRS) but at conventional field strengths, there is difficulty distinguishing glutamate from its precursor and metabolite, glutamine. For this reason, a composite measure called ‘Glx’ is often employed (Yüksel and Öngür 2010). Some, but not all, previous studies in AN participants have reported low Glx levels in frontal brain regions (Ohrmann et al. 2004; Castro-Fornieles et al. 2007; Joos et al. 2011; Blasel et al. 2012) such as anterior cingulate cortex. The availability of MRS at 7 Tesla (7 T) permits clear separation of glutamate and glutamine resonances as well as a more precise quantification of neurometabolites (Tkáč et al. 2009).

The aim of the present study was to use high field MRS to assess brain glutamate concentrations in patients with AN. We selected three voxels for this purpose, occipital cortex (OCC), anterior cingulate cortex (ACC) and putamen (PUT). The OCC is often used for MRS studies because of the lack of field inhomogeneity in this brain region. The OCC has not been linked to anorexia nervosa but elevated levels of glutamate have sometimes been reported in OCC in depressed patients (Yüksel and Öngür 2010). The ACC and PUT (the latter as part of the striatum) have been implicated in the neural circuitry underpinning AN (Kaye et al. 2009).

## Methods

### Participants

All participants gave full written informed consent to the study which was approved by the National Research Ethics Service Committee, South-Central Oxford C. We obtained usable MRS data from 13 female participants (mean age 29.1 years, range 18–41 years) with longstanding AN (mean duration 12.7 years, range 3–26 years) as defined by the Diagnostic and Statistical Manual of Mental Disorders Fifth Edition (DSM-5) and 12 healthy female controls (27.3 years, range 20–41 years). Exclusion criteria for the AN participants were psychosis or substance dependence, contraindication to MRS imaging, pregnancy or breastfeeding, or history of claustrophobia. Similar exclusion criteria were applied to the healthy controls with the addition of any current or past history of psychiatric disorder on DSM-5 and concurrent psychotropic medication.

Ten of the AN participants had current additional current diagnoses of major depression and one also had panic disorder. The mean score of the AN participants on the Eating Disorder Examination Questionnaire (EDE-Q) was 3.7 (range 0.8–6.1) and on the Hamilton Rating Scale for Depression (HAM-D) 20.7 (range 3–36). The corresponding values for the controls were EDE-Q, 0.5 (0.06–1.38) and HAM-D, 0.5 (0–3). The mean BMI of the AN participants was 15.3 (range 13.3–18.2) and that of the controls was 21.3 (range 18.6–25.3). Eight of the AN participants were inpatients and eight were taking psychotropic medications, including mirtazapine 15 mg, fluoxetine 20 and 40 mg, sertraline 100 and 200 mg, citalopram 40 mg, bupropion 75 mg, paroxetine 40 mg, olanzapine 5 and 10 mg, lorazepam 2.5–5 mg, zopiclone 7.5 mg and melatonin 3 mg.

### Magnetic resonance spectroscopy

**Fig. 1:**
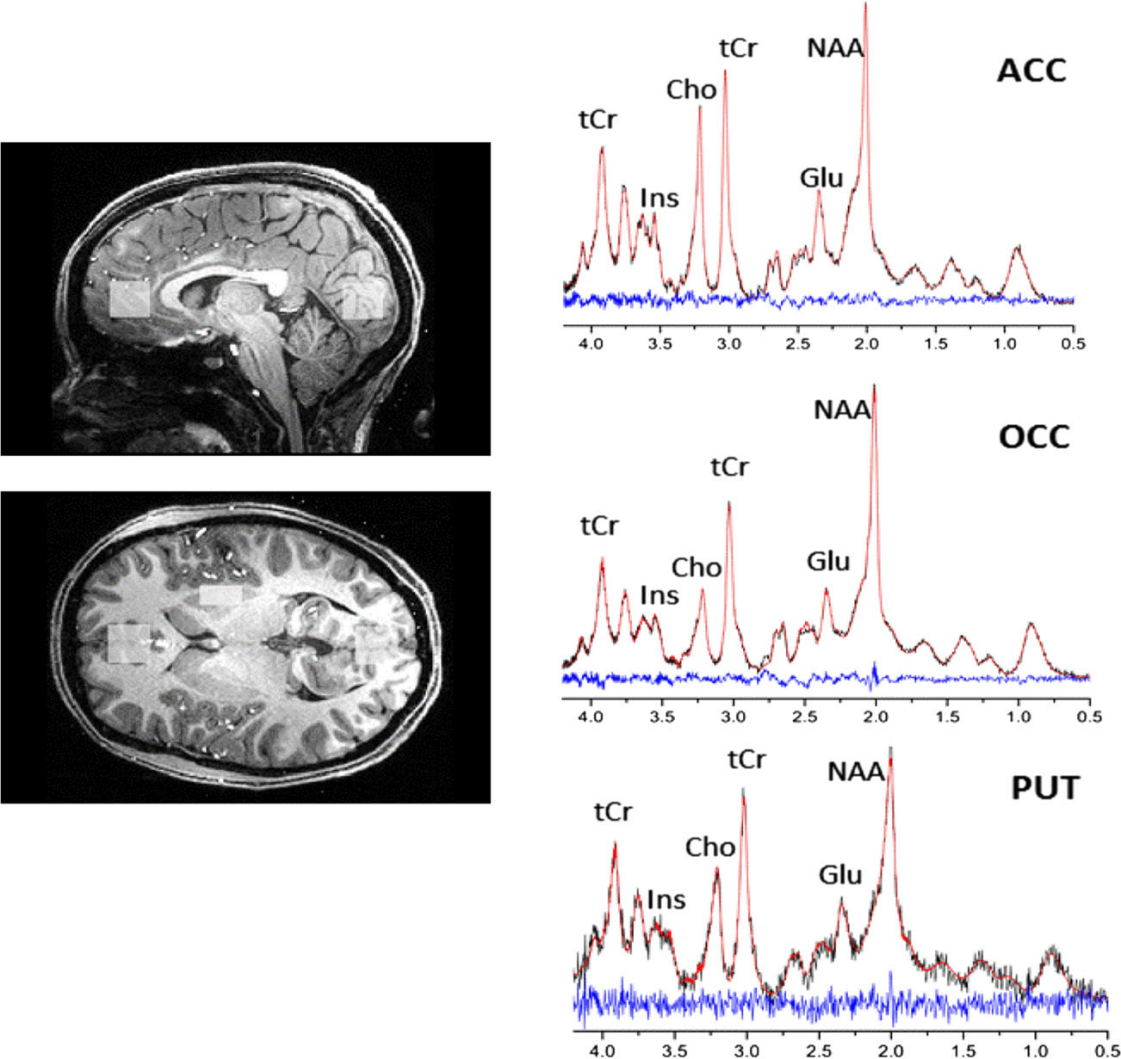
Voxel placement and representative spectra from the anterior cingulate cortex (*ACC*), occipital cortex (*OCC*) and putamen (*PUT*). Each acquired spectrum (64 averages) is overlaid with the metabolite fit from LCModel (*red line*) with major peaks labeled. The difference between the metabolite fit and underlying spectrum is shown below as a residual, which remains small and uniform indicating a high quality spectral fit. *tCR* total creatine, *Ins myo*-Inositol, *Cho* choline, *Glu* glutamate, *NAA* N-acetylaspartate

Participants underwent proton (^1^H) MRS scanning at the Functional Magnetic Resonance Imaging of the Brain (FMRIB) Centre in Oxford. Scanning was performed on a 7-T Siemens MAGNETOM scanner (Siemens, Erlangen, Germany) equipped with a Nova Medical 32 channel receive array head coil. Spectra were measured from three voxels, in the ACC (20 × 20 × 20 mm), in the OCC (20 × 20 × 20mm) and in the PUT (10 × 16 × 20mm) (Fig. 1). Voxels were positioned manually by reference to 1-mm isotropic T1-MPRAGE image. First- and second-order shims were first adjusted by gradient-echo shimming (Shah et al. 2009). The second step involved only fine adjustment of first order shims using FASTMAP (Gruetter and Tkáč 2000). Spectra were acquired using a stimulated echo acquisition mode (STEAM) pulse sequence (TE = 11 ms, TR = 5 s, number of transients = 64) with variable power radiofrequency pulses with optimised relaxation delays (VAPOR) water suppression and outer volume saturation (Emir et al. 2012). Unsuppressed water spectra acquired from the same voxel were used to remove residual eddy current effects and to reconstruct the phased array spectra.

Metabolites were quantified using LCModel (Provencher 2001). The model spectra of aspartate (Asp), ascorbate/vitamin C (Asc), glycerophosphocholine (GPC), phosphocholine (PC), creatine (Cr), phosphocreatine (PCr), γ-amino-butyric acid (GABA), glucose (Glc), glutamine (Gln), glutamate (Glu), glutathione (GSH), *myo*-inositol (*myo*-Ins), N-acetylaspartate (NAA), N-acetylaspartylglutamate (NAAG), phosphoethanolamine (PE), *scyllo*-inositol (*scyllo*-Ins) and taurine (Tau) were generated based on previously reported chemical shifts and coupling constants (Govindaraju et al. 2000; Tkáč 2008) by using GAMMA/PyGAMMA simulation library of VESPA for carrying out the density matrix formalism [VErsatile Simulation, Pulses and Analysis 9]. Simulations were performed with the same RF pulses and sequence timings as that on the 7-T system. A macromolecule spectrum acquired from the OCC, using an inversion recovery sequence (TR = 3 s, TE = 11 ms, inversion time TI = 0.685 s), was included in the model spectra. Metabolite concentrations were obtained relative to an unsuppressed water spectrum acquired from the same VOI assuming a water content of 82% for occipital cortex and anterior cingulate and 78% for the putamen (Gelman et al. 2001).

The MPRAGE images were segmented using FAST (FMRIB’s Automated Segmentation Tool, part of the FSL toolbox) to determine CSF fraction (fCSF) in the voxels (Zhang et al. 2001). Concentrations were then corrected for CSF fraction with the following formula: [Mcorr] = [M]. (1/[1 − fCSF]), where [Mcorr] = corrected concentration and [M] = metabolite concentration from LCModel output.

Metabolites quantified with Cramer-Rao lower bounds (CRLB), estimated error of the metabolite quantification >30% were classified as not detected. As a secondary filter to select reliable metabolite concentrations, only metabolites quantified with CRLB ≤30% in at least half of the spectra from a brain region were reported (see Table 1 for mean CRLB values for each metabolite included in the analysis). If the correlation between two metabolites was consistently high (correlation coefficient <−0.5) in a given region, their sum was reported, such as NAA + NAAG (tNAA, total NAA) and Cr + PCr (tCr, total creatine).

**Table 1:** Magnetic resonance spectroscopy (MRS) measures (μmol/g) and the Cramer–Rao lower bound (CRLB) (%) for glutamate, glutamine, total N-acetylaspartate (tNAA), total creatine (tCr), γ-aminobutyric acid (GABA), inositol and glutathione (GSH) concentrations

	Patients with AN Mean (SEM)	Healthy controls Mean (SEM)	*p* value
Glutamate (ACC) CRLB	10.6 (0.24) 2.1 (0.6)	11.6 (0.19) 2.2 (0.7)	0.004
Glutamate (OCC) CRLB	8.7 (0.15) 2.3 (0.7)	9.4 (0.24) 2.1 (0.6)	0.029
Glutamate (PUT) CRLB	7.6 (0.29) 4.5 (1.3)	8.3 (0.18) 3.9 (1.4)	0.058
Glutamine (ACC) CRLB	3.5 (0.16) 7.0 (2.0)	3.20 (0.24) 7.6 (2.3)	0.28
Glutamine (OCC) CRLB	2.90 (0.12) 6.7 (1.9)	2.62 (0.08) 7.7 (2.2)	0.06
Glutamine (PUT) CRLB	2.52 (0.21) 14.9 (4.3)	2.27 (0.12) 15.1 (4.6)	0.31
tNAA (ACC) CRLB	11.09 (0.30) 1.5 (0.4)	11.68 (0.29) 1.4 (0.4)	0.17
tNAA (OCC) CRLB	13.62 (0.37) 1.0 (0.3)	13.48 (0.35) 1.2 (0.4)	0.79
tNAA (PUT) CRLB	9.01 (0.26) 2.6 (0.7)	9.28 (0.21) 2.4 (0.71)	0.43
tCr (ACC) CRLB	9.01 (0.33) 1.8 (0.5)	9.16 (0.24) 2.0 (0.6)	0.72
tCr (OCC) CRLB	8.78 (0.28) 1.7 (0.5)	8.61 (0.14) 1.8 (0.5)	0.62
tCr (PUT) CRLB	5.01 (0.30) 2.8 (0.8)	4.89 (0.40) 2.6 (0.8)	0.81
GABA (ACC) CRLB	1.98 (0.14) 11.1 (3.2)	2.20 (0.70) 8.1 (2.6)	0.29
GABA (OCC) CRLB	2.01 (0.12) 8.7 (2.5)	1.70 (0.11) 12.2 (3.5)	0.07
GABA (PUT) CRLB	2.21 (0.67) 11.5 (3.5)	2.12 (0.09) 10.8 (3.3)	0.57
Inositol (ACC) CRLB	6.50 (0.44) 3.4 (1.0)	7.78 (0.27) 3.1 (0.9)	0.022
Inositol (OCC) CRLB	5.39 (0.38) 3.7 (1.1)	6.83 (0.16) 2.9 (0.8)	0.002
Inositol (PUT) CRLB	3.94 (0.37) 9.1 (2.6)	4.45 (0.16) 5.6 (1.7)	0.23
GSH (ACC) CRLB	1.19 (0.07) 10.2 (2.9)	1.27 (0.10) 8.9 (2.8)	0.38
GSH (OCC) CRLB	0.95 (0.03) 10.5 (3.0)	0.94 (0.04) 10.67 (3.08)	0.85
GSH (PUT) CRLB	1.51 (0.45) 15.0 (4.5)	1.10 (0.05) 15.4 (4.6)	0.43

*ACC* anterior cingulate cortex, *OCC* occipital cortex, *PUT* putamen

### Statistics

Statistical analyses were performed in SPSS version 22. Differences in metabolite concentrations between AN participants and controls were determined using repeated measures analysis of variance (ANOVA), with ‘region’ (ACC vs PUT vs OCC) as a within-subject factor and ‘diagnosis’ (AN vs control) as a between subject factor. Significant interactions were followed up with unpaired *t* tests (two tailed). Correlations were carried out using Pearson’s product moment.

## Results

**Fig. 2:**
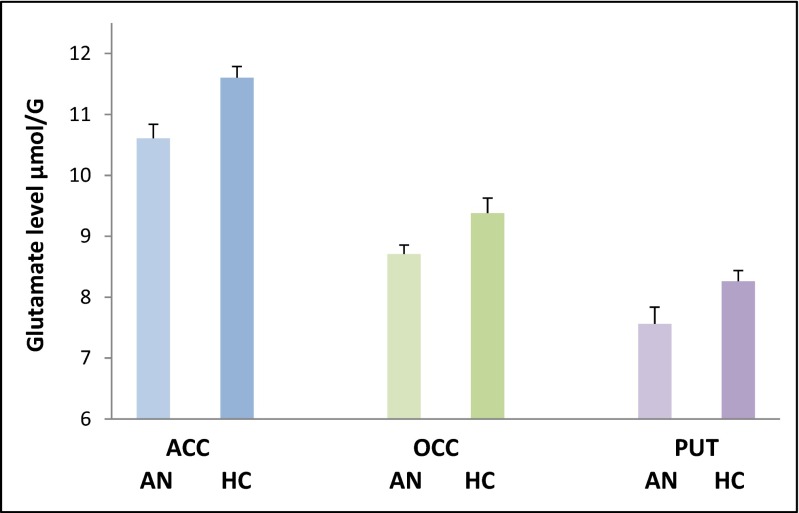
Mean (SEM) glutamate levels in 11 female patients with anorexia nervosa (AN) and 10 female healthy controls (CON) in three brain regions, anterior cingulate cortex (*ACC*), occipital cortex (*OCC*) and putamen (*PUT*). There is a main effect of diagnosis on glutamate levels, independent of region (*F* = 13.7; df = 1.19; *p* = 0.002)

Four MRS spectra (two for the ACC, one for the OCC and PUT) were excluded due to their quality, leaving 11 AN participants and 10 controls who had valid glutamate and glutamine data for all three brain regions The repeated measures ANOVA for glutamate showed a main effect of diagnosis (*F* = 13.7; df = 1.19; *p* = 0.002) but no interaction with region (*F* = 0.053; df = 2.19; *p* = 0.95) indicating a reduction of glutamate in AN participants in all three voxels (Fig. 2). In contrast, there was no main or interactive effect of diagnosis on glutamine levels (*F* = 2.99; df = 1.19; *p* = 0.10 and *F* = 0.023; df = 1.19; *p* = 0.88) (Table 1). Consistent with these findings, there was a significant main effect of diagnosis on the ratio of glutamine to glutamate (*F* = 6.28; df = 1.19; *p* = 0.021) with higher ratios in the AN participants.

Similar analyses of other neurometabolites showed no significant main or interactive effects of diagnosis on total N-acetylaspartate (NAA), total creatine, GABA, or glutathione. There were, however, a significant main effect of diagnosis and a significant diagnosis by region interaction for inositol (*F* = 7.62; df = 1.19; *p* = 0.012 and *F* = 4.07; df = 2.38; *p* = 0.025, respectively) where inositol levels in ACC and OCC were lower in AN participants (see Table 1). In the AN participants, there was no significant correlation between glutamate level in any of the brain regions studied and total score on the EDE-Q and HAM-D (all *p* values >0.1).

## Discussion

Because of the small numbers of participants studied, this study should be regarded as a pilot investigation. Within this limitation, we found that glutamate levels in AN participants were diminished in all three voxels studied. Interestingly, concentrations of the glutamate precursor and metabolite, glutamine, were not significantly changed which resulted in a significantly increased ratio of glutamine to glutamate in AN. This suggests that the conversion of glutamine to glutamate in glutamate neurons is diminished in AN (Yüksel and Öngür 2010). It is also possible, however, that the availability of glutamate might be generally decreased if amino acids are being used as an energy source in AN patients, though this does not seem to be the case from measures of amino acids in plasma in AN (Moyano et al. 1998). A number of lower field strength MRS studies have examined Glx levels in anterior brain regions in AN with conflicting results; however, some have reported diminished Glx concentrations (Ohrmann et al. 2004; Castro-Fornieles et al. 2007; Joos et al. 2011; Blasel et al. 2012). Our study suggests that diminished glutamate is the key contributor to this decrease in Glx.

Many neurobiological changes have been reported in AN patients relative to healthy controls and such changes often reflect the effects of weight loss (Phillipou et al. 2014). The same could clearly be true of the lowered brain glutamate levels we have observed. Further studies will be needed in recovered patients as well as those at high risk of AN to determine whether lowered glutamate activity might play a pathophysiological role in AN. The participants in this study were at different stages of treatment and were taking a variety of medications. It will be important that future work attempts to study participants with less variation in these factors.

It is also commonly reported that AN patients have decreased cortical grey matter volume which could be associated with altered levels of brain neurochemicals (Phillipou et al. 2014). However, we took this possibility into account by correcting for CSF volume in the voxel. Also, we found no reduction in AN in total NAA which is believed to be an important marker of grey matter integrity (Birken and Oldendorf 1989). MRS glutamate levels can fluctuate with the menstrual cycle (Batra et al. 2008). Hence, it is also possible that the changes we have seen reflect alterations in sex steroid levels in the AN participants. In this context, it is important to note that we studied female participants only and although the incidence of AN in males is generally regarded as about tenfold less than that in women, under-diagnosis in men may occur (Raevuori et al. 2014). Clearly, we are not able to extrapolate our MRS findings in women to men with AN.

AN patients frequently suffer from comorbid depression and major depression has been associated with lowered Glx levels, particularly in anterior brain regions (Yüksel and Öngür 2010; Luykx et al. 2012). Thus, accompanying depressive symptomatology is a possible explanation for lowered glutamate in AN. Against this, in depressed patients, Glx levels in OCC have been reported as either normal or increased (Yüksel and Öngür 2010) whereas in the AN patients the reduction in glutamate also involved the OCC. Further, there was no correlation in the AN participants between depression severity (measured by the HAM-D) and glutamate level. As is common in the treatment of severe AN, many participants were also taking psychotropic medication, principally selective serotonin reuptake inhibitors (SSRIs). However, in a sample of depressed patients, we found no effect of SSRI treatment on MRS glutamate measures (Godlewska et al. 2015) which makes concomitant treatment with SSRIs an unlikely explanation for our findings. However, other patients were taking different kinds of psychotropic medication and in future work it will be important to study AN participants who are medication free.

The use of MRS at 7 T also permitted the accurate measurement of several other neurometabolites. With one exception, there was no difference between AN participants and healthy controls. However, there was a decrease in inositol in AN in two of the three brain regions studied. We made no correction for multiple comparisons and therefore this finding must be treated with particular caution. However, some, but not all, previous MRS studies have also reported lower levels of inositol in cortical regions in AN patients (Roser et al. 1999; Ohrmann et al. 2004; Castro-Fornieles et al. 2007; Joos et al. 2011; Blasel et al. 2012). These investigations have linked the low inositol levels to the metabolic effects of starvation or to concomitant depression (Roser et al. 1999; Ohrmann et al. 2004).

In conclusion, our study is the first, as far as we are aware, to measure glutamate in AN with MRS at 7 T and demonstrates what appears to be a rather generalised reduction in brain glutamate levels. Further work will be needed to assess whether treatments manipulating brain glutamate levels might be of value in AN management.

## Electronic supplementary material

ESM 1. (DOCX 13 kb)
