# Supplementary material for: Brain glutamate in anorexia nervosa: a magnetic resonance spectroscopy case control study at 7 Tesla
Source: Psychopharmacology (Berl). 2016 Dec 1;234(3):421–6. doi: 10.1007/s00213-016-4477-5 (PMC5225214; doi:10.1007/s00213-016-4477-5)
Supplement: Supplementary file 1 — (DOCX 13 kb) [file 213_2016_4477_MOESM1_ESM.docx]

|  | Patients with AN | Healthy controls |
| --- | --- | --- |
| S/N (ACC) | 30.7 (8.9) | 35.6 (10.8) |
| S/N (OCC) | 46.9 (13.5) | 43.5 (12.6) |
| S/N (PUT) | 13.1(3.6) | 15.3 (4.6) |
| FWHM (ACC) | 0.041 (0.012) | 0.034 (0.010) |
| FWHM (OCC) | 0.034 (0.010) | 0.033 (0.009) |
| FWHM (PUT) | 0.053 (0.015) | 0.052 (0.016) |

Supplementary Table 1: Measures of spectral quality. S/N= signal to noise ratio; FWHM= full-width at half maximum; ACC – anterior cingulate cortex, OCC – occipital cortex, PUT - putamen
